# Ligand field tuning of *d*-orbital energies in metal-organic framework clusters

**DOI:** 10.1038/s42004-023-00863-z

**Published:** 2023-04-12

**Authors:** Brian G. Diamond, Lillian I. Payne, Christopher H. Hendon

**Affiliations:** Department of Chemistry and Biochemistry, University of Oregon, Eugene, OR 97403 USA

**Keywords:** Crystal field theory, Computational chemistry, Metal-organic frameworks

## Abstract

Linker functionalization is a common route used to affect the electronic and catalytic properties of metal-organic frameworks. By either pre- or post-synthetically installing linkages with differing linker moieties the band gap, workfunction, and exciton lifetimes have been shown to be affected. One overlooked aspect of linker functionalization, however, has been the impact on the metal *d*-orbital energies to which they are bound. The ligand field differences should result in substantial changes in *d*-splitting. In this study we use density functional theory (DFT) to study the energetics of *d*-orbital energy tuning as a function of linker chemistry. We offer a general descriptor, linker pK_a_, as a tool to predict resultant band energies in metal-organic frameworks (MOFs). Our calculations reveal that simple functionalizations can affect the band energies, of primarily metal *d* lineage, by up to 2 eV and illustrate the significance of this band modularity using four archetypal MOFs: UiO-66, MIL-125, ZIF-8, and MOF-5. Together, we show that linker functionalization dramatically affects *d*-energies in MOF clusters and highlight that linker functionalization is a useful route for fine-tuning band edges centered on the metals, rather than linkers themselves.

## Abstract (web summary)

Linker functionalization is a commonly used strategy to affect the electronic and catalytic properties of metal–organic frameworks, but its impact on the *d*-orbital energies of the metals to which the linkers are bound has been largely overlooked. Here, DFT calculations are used to study the energetics of *d*-orbital energy tuning as a function of linker chemistry in UiO-66, MIL-125, ZIF-8 and MOF-5.

## Introduction

MOFs are of increasing interest in catalysis because their molecular level tunability is a feature that has proven useful in photocatalytic applications where careful control of frontier band potentials are required^1–3^. The ability to affect both the band gap and nature of the transition—e.g., metal-to-ligand band parentages—play critical roles in the population and lifetimes of excitons, respectively. There exist several routes to creating and modifying MOF catalysts, including linker functionalization and transmetalation^4–6^. To date, linker functionalization has been largely portrayed to affect the energetics of the linker, and subsequently the linker-based bands in MOFs. The interplay between ligand electronics and metal *d*-splitting has remained an overlooked and fundamental aspect of MOF chemistry, and the ability to fine-tune-specific bands remains a grand challenge and target for MOF-based catalyst development.

The electronic dependence on composition has been investigated by both theory and experiment, with the aim of developing design principles that can be used to predict material properties pre-synthesis. For example, studies have shown that the electronic structure of the isolated linker is a reasonable predictor for the electronic structure of the MOF^5,7–9^. This result is curious because there are a variety of possible frontier band compositions between MOF structures. The four MOF types are those with, (I) organic valence and conduction bands (e.g., Zr-UiO-66^10^, Zn-MOF-5^11^), (II) inorganic valence/organic conduction bands (e.g., Zn-NDI^12^, but these are rare because it is difficult to design a linker that is so readily reduced), (III) inorganic valence/inorganic conduction bands (e.g., Cu-HKUST-1^13^), and (IV) organic valence/inorganic conduction bands (e.g., Ti-MIL-125^14^). For materials featuring organic valence bands (type I and IV), it is well-established that linker functionalization affects the valence band energy and material ionization potential. For example, the addition of the electron donating group –NH_2_ reduces the electronic band gap in benzene dicarboxylate (bdc)-containing MOFs by ~1.3 eV^4,9^. It is decidedly more difficult to affect the valence band energies of systems that have inorganic extrema, simply because there are fewer chemical handles available.

In a 2020 study, Syzantseva and co-workers showed that the free-bdc linker orbitals were good energetic estimates of the MOF-bound linker electron energies^7^. Hamad and co-workers showed a similar trend for zeolite imidazole frameworks (ZIFs) where the HOMO/LUMO gap of the linker is correlated to the band gap of the resulting MOF^5^. A combined experimental and computational study by Hemelsoet and co-workers showed that in UiO-66, a MOF whose band edges are primarily linker character, the optical absorption across the HOMO/LUMO of the linker is related to the optical band gap absorption of the MOF^15^. These electronic structure studies are instrumental in better understanding the photophysical properties of MOFs, but none have thus far taken into account the linker’s effect on the metal *d*-orbital energies.

Considering the four types of energy alignments, one overlooked aspect of linker functionalization—independent of the MOF frontier band parentage—is the affect that linker functionalization has on *d*-orbital splitting, i.e., the ligand field effects. Since the *d*-orbital energies are highly sensitive to changes in ligand field, changes in ligand electron density should influence the stability and energetics of metal *d-*states. Therefore, linker functionalization should provide a unique avenue to fine-tuning valence band *d*-orbitals in type II and III MOFs, and conduction band *d*-orbitals in type III and IV materials.

**Fig. 1:**
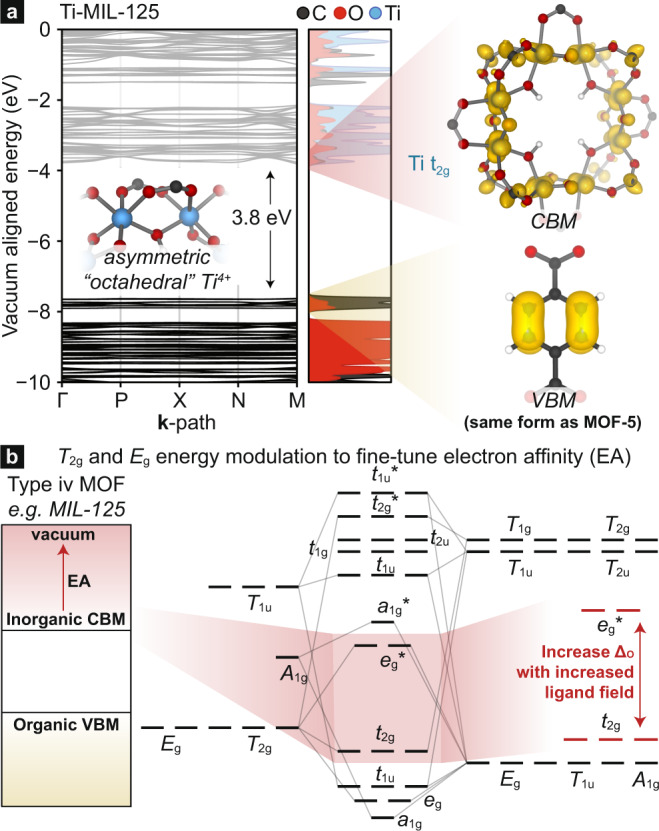
The electronic structure of Ti-MIL-125. Ti-MIL-125 features pseudo-octahedral Ti^4+^ ions. In complex MOF clusters, such as that of MIL-125, **a** the valence band may have organic π-antibonding character, whereas the conduction band is delocalized around the Ti-octamer. **b** In the octahedral configuration the sigma manifold affects the energy of the *e*_g_* and the ligand LUMOs control the stability of the metal *t*_2g_ orbitals. The Ti-orbitals have d_xy/xz/yz_ character, and hence should be affected by the π-manifold of the linker.

The application of high-symmetry splitting diagrams are useful predictive tools for transition metal energies, even when the metal coordination environment diverges from the high-symmetry assignments (e.g., the Zn^2+^ ions in the MOF-5 cluster are locally tetrahedral but are formally C3v, Fig. S1; the Ti^4+^ centers in MIL-125 are locally octahedral, but are formally C1, Fig. 1a). There are a variety of computational methods one can use to probe the effective ligand field. The most common method involves the calculation of a model octahedral or tetrahedral transition metal complex, and *d*-splitting is an indicator of relative ligand field strength^16–21^. One can also use charge parsing schemes to assess donor atom charge. The issue with parsing methods is that linker/metal interactions are not limited to σ-donation. For example, Bader charges or other approaches do not directly probe unoccupied orbital contributions (e.g., ligand *t*_2_ linker orbitals in tetrahedral systems). Alternatively, the free-linker pK_a_ is a useful descriptor for *d*-splitting and subsequent band modularity because pK_a_ is a property that depends on the cumulative electronic structure of the Lewis base. Here, we demonstrate that the bands with metal *d* character can be affected by up to 2 eV depending on linker functionality. We rationalize these energetic perterbations from ligand field theory. Our approach is general and predictive design principle for fine-tuning *d*-band energies in MOFs and can be used to target valence and conduction band energy manipulation.

## Results and discussion

Inevitably, we will show that pK_a_ is a useful predictor of *d*-orbital energy splitting and hence a valuable approach to fine-tune metal-band energies. However, to understand this relationship we must first discuss the orbitals that should be affected by ligand functionalization. From ligand field theory, a metal in a perfectly tetrahedral or octahedral environment should have their *t*_2_ and *t*_2g_ energy levels (i.e., metal *d*_xy/xz/yz_) affected by the π-ligand manifold, respectively. An octahedral splitting diagram with the qualitative inclusion of the ligand π-manifold is shown in Fig. 1b, while a similar tetrahedral splitting diagram can be found in Fig. S1. In octahedral systems, the *t*_2g_-to-*e*_g_* gap depends on the σ contribution from the ligand, in addition to whether the ligand can π-donate or accept. Since most MOFs are formed with metal-carboxylates, and carboxylates are generally thought to be σ-donors as well as both π-donors and acceptors (since the p_z_ are half-filled), the *t*_2g_ orbital energy should be elevated with increasing electron density on the carboxylates. However, it is not immediately clear whether the donor or acceptor character is dominant in MOF linkers and will indeed be elucidated with computation. For now, let us assume that carboxylates are weakly π-donating, and functional groups that increase the electron density around the carboxylate should serve to push the *t*_2g_ orbitals towards the vacuum level. Similarly, the *e*_g_ energy should be determined primarily by the σ contribution from the ligand, where increased σ density elevates the *e*_g_ towards the vacuum level.

**Fig. 2:**
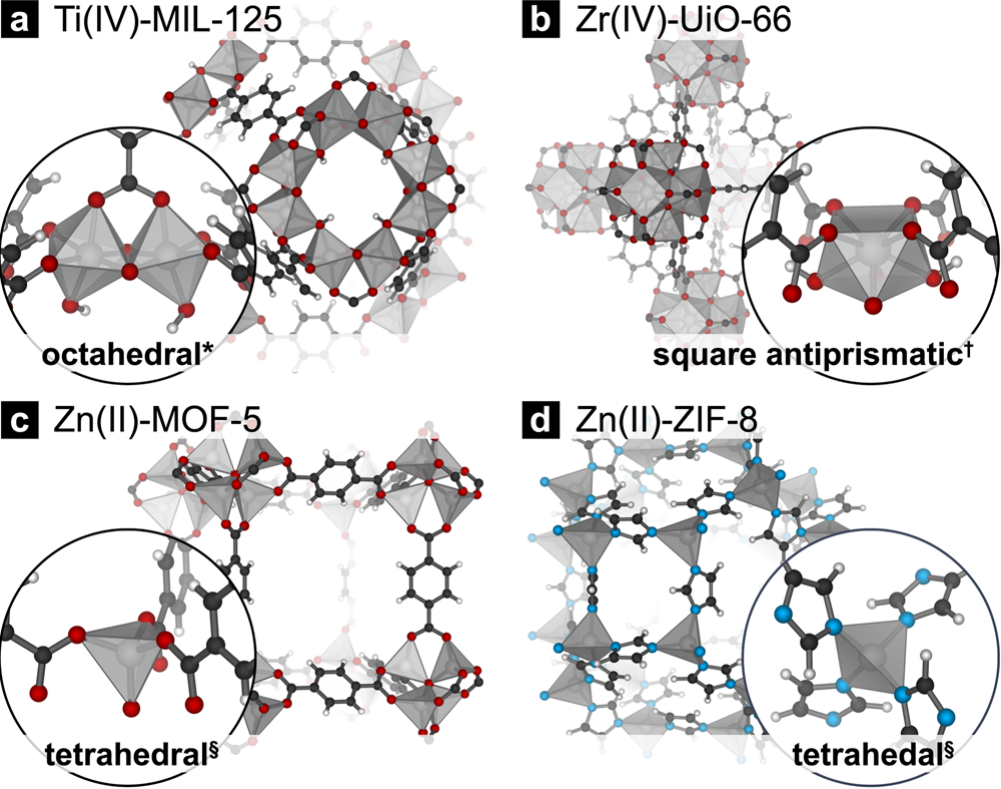
An examination of the metal coordination environments in four common MOFs. Four MOFs considered in this ligand functionalization study. **a** Ti-MIL-125 features octameric Ti(IV)-centers in (*pseudo)octahedral environments. **b** Zr-UiO-66 features 7-coordinate Zr(IV) centers in (^†^pseudo)square antiprismatic coordination. **c** Zn-MOF-5 is constructed from (^§^pseudo)tetrahedral Zn(II) centers that form a super tetrahedral Zn_4_O cluster. **d** Zn-ZIF-8 features (^§^pseudo)tetrahedral Zn(II) centers, only lowered in symmetry by the polarization of the linker.

With this textbook analysis of orbital energies in mind we can now consider the local coordination environments of metals in MOF clusters, Fig. 2. For this study we selected four familiar frameworks, Zr-UiO-66, Zn-MOF-5, Ti-MIL-125, and Zn-ZIF-8. These frameworks allow us to make comparisons between MOFs with the same metal but different ligands, the same ligand with different metals, and different coordination environments. An important aspect in the electronic applications of MOFs is the character of the band edges. MIL-125, a type IV d^0^ MOF, with a titanium centered conduction band, and each metal center connects to three unique organic ligands. The other three inner-sphere ligating ligands are *µ*_*2*_-OH and *bis*-*µ*_*2*_-O, Fig. 2a. The other MOFs are type I (ZIF-8, UiO-66, and MOF-5), with linker character at both extrema. UiO-66 also features d^0^ Zr(IV) with four bdc-carboxylates and two *µ*_*3*_-OH and *µ*_*3*_-O Fig. 2b. Both ZIF-8 and MOF-5 contain d^10^ Zn(II); MOF-5 features Zn(II) with three bdc-connections, and a single *µ*_*4*_-O (Fig. 2c), while ZIF-8 Zn(II) are ligated by four imidazolates (Fig. 2d). Subsequent linker functionalization should affect the conduction band *d*-orbitals in UiO-66 and MIL-125, and the valence band in ZIF-8 and MOF-5. The same functionalization will also affect the chemical properties of the free linker itself, such as its pK_a_ and electron energies.

In Pearson’s seminal paper^22^, he demonstrated that Lewis “hardness”^23^ could be estimated from the energy of the molecular HOMO and LUMO, aligned to the vacuum level, an example is shown in Fig. S2. Thus, a DFT calculation of functionalized linkers should provide a direct estimate of its hardness, which is related to ligand field. pK_a_ is also dependent on hardness, because the Brønsted acidity of the linker is related to the Lewis basicity of the conjugate base, and the pK_a_ may be calculated through a simple thermodynamic cycle by optimizing the structures of the protonated and deprotonated linker and calculating their energies in the gaseous and (pseudo)solvated phases^24^.

**Fig. 3:**
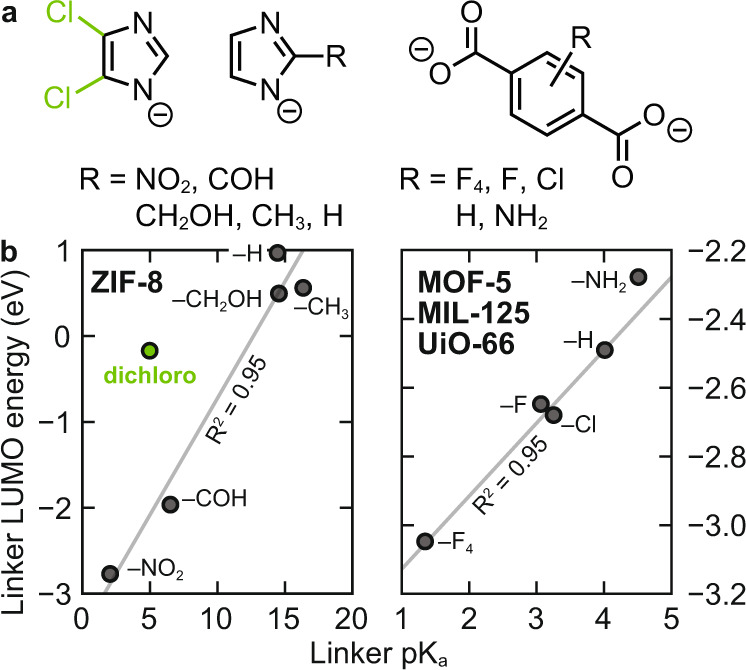
The relationship between linker pKa and LUMO energy. **a** Functionalized ligands considered in this study. **b** The computed pK_a_ is a good descriptor for the neutral linker LUMO (i.e., their electron affinity). The trend is linear (R^2^ = 0.95) for functional groups appended to either the C_1_ position on the imidazole, or any position on the aromatic benzene dicarboxylate. Dichloro-functionalized imidazole is relatively acidic but has an unusually high LUMO energy, as the chloro groups withdraw density from the C = C bond, which comprises the LUMO (see Fig. S3).

By plotting the computed electron affinity of the acidic linkers (i.e., LUMO energies for molecules presented in Fig. 3a), and their respective pK_a_, we find there is a linear relationship between the two, Fig. 3b. Generally, the presence of an electron withdrawing group stabilizes the conjugate base, stabilizing the electron density of the anion. Additionally, the functionality results in a large range of predicted pK_a_ values for functionalized imidazoles (~2–~16 for –NO_2_ and –CH_3_ functionalities, respectively), and bdc derivatives (~1.5–4.5, for –F_4_ and –NH_2_ functionalities, respectively). These calculations provide the basis for our hypothesis that the linker pK_a_ should be a good descriptor for ligand field strength because it is a property that depends on the ligand electronics.

However, the carboxylate-H^+^ bond strength (related to the pK_a_) is in essence a measure σ-density, which formally should not be related to the effect on the band edges in any of these MOFs. This is because the conduction bands in UiO-66/MIL-125 should comprise, among others, the π-donating orbitals on the linker, whereas the MOF-5/ZIF-8 valence bands should comprise the linker’s π-accepting orbitals. Thus, we highlight that the relationship between pK_a_ and predicted band energy in the MOF is a descriptor that conflates several fundamental chemical properties of the linker, and is linearly related to the linker LUMO energy, Fig. 3b.

We sought to monitor the *d*-orbital energies of the band extrema as a function of linker functionality and relate the band energies to the linker pK_a_. Critically, to make the comparison the MOF electron energies must be aligned to the vacuum level. This way we can observe the effect of functionalization on metal *d*-orbital energies relative to one another. Using our code STREUSEL^25^, we can align the MOF valence band to the vacuum level using a previously described method^26^. From there, we can plot a density of states, and use the density to direct us to the frontier metal orbitals. For example, Fig. S4 shows the density of states of the ZIF-8 with different linker functionalization. The valence band energy shifts with linker functionalization, and is linker centered at its extrema. The Zn *d*-states also shift and can be seen moving around slightly deeper in the density of states. Additionally, the band gap of ZIF-8 was calculated to be 5.3 eV, which matches closely with reported values from UV-Visible measurements^27^. In general, this band gap is reduced as there is more electron withdrawing functionalization on the linker. A similar trend is observed for the other MOFs, Table. S1.

**Fig. 4:**
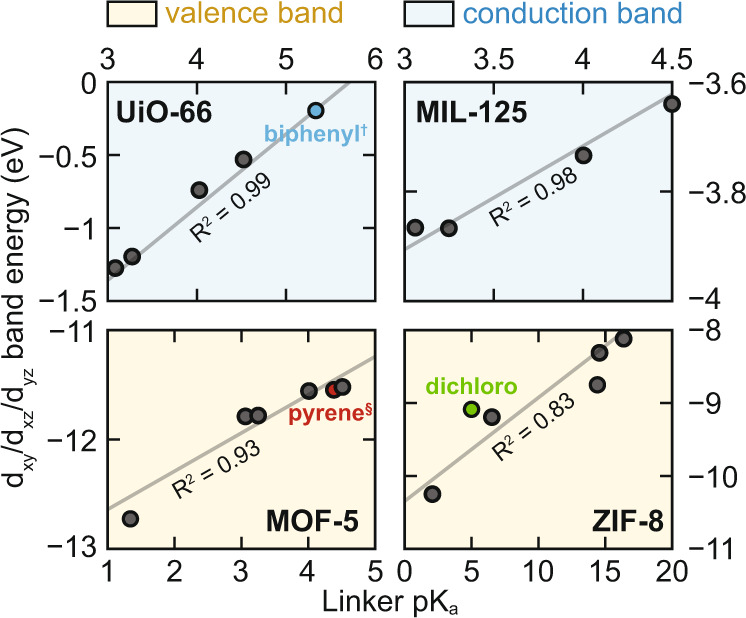
The relationship between linker pKa and MOF d-orbital energy. The relationship between free-linker pK_a_ and *d*-orbital extracted from the DFT computation of the bulk functionalized MOF. The *d*-energies are linearly related to the linker pK_a_ with strong positive correlation. Dichloro functionality in the C_2,3_ position is highlighted in green. In blue, biphenyl^†^ is substituted for the aromatic portion of bdc (i.e., biphenyldicarboxylate) and this MOF is also known as UiO-67. In red, pyrene^§^ is substituted for the aromatic portion of bdc (i.e., pyrenedicarboxylate) and this MOF is also known as IRMOF-14^36^. The gradients are 0.49 (UiO-66), 0.38 (MOF-5), 0.16 (MIL-125), and 0.13 (ZIF-8) eV/pK_a_. Bands that show up in the conduction band (d^0^ MOFs) are labeled with a blue background, while those that appear within the valence band (d^10^ MOFs) are labeled with a yellow background.

To make a quantitative assessment of the extent to which the metal *d*-orbitals are affected we follow a general protocol of searching for metal-character bands that we can easily keep track of (e.g., the d_xy/xz/yz_ in Fig. 1a). By selecting a metal-orbital for each system—d_xy/xz/yz_ in all cases—we can then recover their energy and relate it to the free-linker pK_a_, Fig. 4. There, the plot is divided into two panels; those whose *d*^10^ and hence appear in the valence band (MOF-5 and ZIF-8 derivatives) and those that are *d*^0^ and appear in the conduction band (MIL-125 and UiO-66 derivatives). Each plot contains limited data points, as there are a limited number of functionalized linkers that are stable in a given structure. As such, the numeric values of gradient serve as more of a qualitative comparison, than a quantitative property. Notably, there is a strong linear relationship between free-linker pK_a_ and *d*-energy in the MOF, with the most dependence occurring for UiO-66 and MOF-5 (0.49 and 0.38 eV/pK_a_, respectively). MIL-125 and ZIF-8 are like one another with gradients of 0.16 and 0.13 eV/pK_a_, respectively. The difference in gradient is to be expected, as each of the MOFs have different coordination environments and these data point to a general observation that metal *d*-orbitals respond to ligand fields differently in different MOFs (there is not one unified gradient that relates linker functionality to *d*-energy change).

**Fig. 5:**
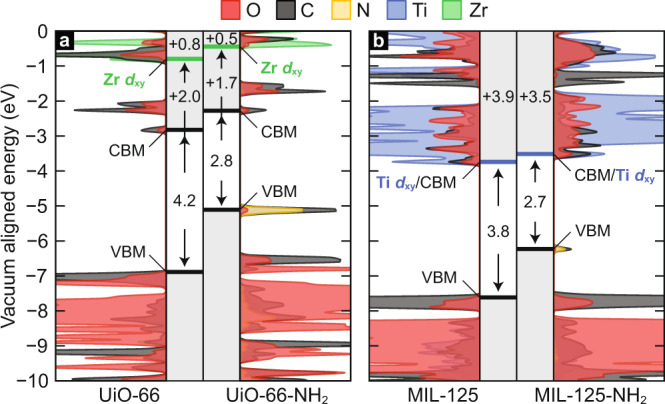
A comparison of the electronic density of state in Ti-MIL-125 and Zr-UiO-66, and their aminated derivatives. Conduction band engineering *d*^0^-MOFs using linker amination. **a** The density of states of reveals UiO-66 is type I with a VBM and CBM defined by organic orbitals. The Zr *d*_xy_ orbitals are 2.0 eV positive of the CBM in non-functionalized UiO-66, sitting at ~−0.75 eV. Amination moves the Zr orbitals 250 meV more positive. **b** MIL-125 is type IV with a VBM and CBM defined by linker and metal orbitals, respectively. The Ti *d*_xy_ orbitals shift 100 meV more positive with amination. In both cases, the aminated MOF features a reduced band gap by >1.0 eV. In the UiO-66 series, the ligand-to-metal transition (VBM-to-green band) shifts from 6.2 to 4.5 eV upon the addition of –NH_2_. The workfunctions are computed by summing the vertical values provided for each MOF.

Given that all slopes are positive, and the orbitals we monitor are d_xy/xz/yz_ in character, we can reinforce the conclusion that less acidic linkers are indeed π-donors as octahedral *d*^0^ metals should accept π-electrons and elevate their *d*-energy in the conduction band, and tetrahedral *d*^10^ should see a similar effect but in the valence band. Perhaps more remarkable is the magnitude of band energy shifting. There is a strong linear relationship between pK_a_ and Zr *d*-energy in UiO-66 and its functionalized derivatives. Unfunctionalized UiO-66 features *d*-orbitals at –0.75 eV from vacuum, and amination results in elevation of those orbitals to –0.55 eV. This is an interesting result because there is ongoing debate in the literature as to the origin of the Zr(III) ESR signal observed^28^ upon photo-irradiation of the aminated material, but not the conventional MOF^29^. We believe that this is because of a more nuanced effect—amination closes the electronic gap from 4.2 to 2.8 eV^30,31^. Since the *d*-states are only shifted by 0.25 eV, but the band gap shifted by 1.4 eV, the resulting ligand-to-metal excitation is decreased from 6.2 eV down to 4.5 eV, well within the UV-Vis., Fig. 5a. It is possible that linker functionalization may also affect linker vacancy population, but it is difficult to imagine a vacancy resulting in a significantly stabilized accessible Zr *d*-state.

In a more subtle case, MIL-125-NH_2_ shows a 100 meV shift in conduction band energy compared to its unfunctionalized analogue, Fig. 5b. Although 100 meV is relatively small compared to shifts achieved for the other MOFs, it should be detectable from solution phase electrochemical measurements, optical measurements and may even affect the catalytic performance of the MOF for hydrogen evolution^32–34^. Moving from MIL-125-NH_2_ to MIL-125-F results in a 200 meV shift in conduction band energy. Considering a previous study from our group^35^, the energy of the conduction band is critical for deliberately disfavoring the hydrogen evolution reaction. Thus, the fluoro-functionalized MOF may be sufficiently stabilizing to turn off hydrogen evolution and increasing H^•^-atom loading the MOF, Fig. 5b.

In MOF-5, each Zn ion is bound to three bdc linkers and functionalization results in up to 1 eV energy shift of the highest energy Zn *d*_xy/xz/yz_ orbitals. In the most extreme case, tetrafluoro-bdc pushes the orbital 900 meV deeper into the valence band. Notably, the orbital energy for MOF-5 and its isoreticular pyrene-based analogue, IRMOF-14^36^, is basically indistinguishable. That is, both Zn ions are deep within the valence bands are redox inactive. This points to a general observation that MOF-5 and isoreticular analogues all feature relatively deep Zn *d*-states and need to be transmetalated to access redox active metal centers^37^.

Finally, of the MOFs examined, the ZIF series shows the least goodness-of-fit to a linear regression, while maintaining a strong positive relationship between linker pK_a_ and Zn *d*-orbital energies. Bracketing the two extremes are methyl-imidazole and nitro-imidazole, with computed pK_a_s of ~16 and ~2, respectively. This vast difference in linker acidity results in dramatic differences of Zn(II) *d*-energies, from −8.0 to −10.2 eV, or a staggering 2.2 eV in ligand field tuning. The residuals are large because the dichloro-functionalized imidazole is functionalized in the C_2,3_ position, which is chemically dissimilar to the other functionalizations (per Fig. 3a). In both Zn(II) cases, the orbitals sit within the valence band and are likely not chemically active for photocatalysis. We rather selected them because they highlight the central point of this study—ligand field effects from linker functionalizations can dramatically affect *d*-orbital energies in MOF clusters.

Together, these data highlight a critical but overlooked aspect of linker chemistry in MOFs: the impact of the linker on the metal *d*-energies. Of course, synthetic chemists have shown that the linker functionality affects the synthetic conditions in which they are formed, presumably due to differences in acidity of the linker. To account for this difference in acidity, creative solutions to MOF syntheses have been developed^38^. But even then, little attention has been paid to the effect of metal energetics as the result of differences in acidity. We believe this is an important and fundamental aspect of MOF chemistry, but one that is costly to compute on periodic bulk MOFs. To circumvent this, we showed that there is a positive relationship between linker pK_a_ and *d*-orbital band energy in four dissimilar MOFs. The same concept will apply beyond type I and IV MOFs examined here and should prove to be a useful design principle in MOF catalyst design. In the most promising examples, we offered one explanation for the observation of Zr(III) *d*-states in photoexcited UiO-66, and in another example we showed that the conduction band energy of MIL-125 can be tuned by 200 meV using common linker chemistries. More broadly, we demonstrated that linker pK_a_ is a valuable descriptor and predictor of *d*-orbital energy in MOFs and will vastly accelerate materials discovery for precisely tuned metal energies.

## Computational methods

All periodic calculations were performed within the Kohn-Sham DFT framework as implemented in Vienna ab initio Simulation Package (VASP 5.4.4)^39^. The lattice parameters and ion positions of all MOFs studied were geometrically optimized using the PBEsol functional^40^, with a 2 × 2 × 2 k-mesh centered at gamma and a 500.00 eV planewave cutoff^41^. The ionic and electronic convergence criteria were 0.005 eV and 10^−5^ eV respectively. The electronic structure of the geometrically optimized MOFs were recovered from an HSE06 single point with the same basis at the gamma point^42,43^. Molecular calculations to determine pK_a_ and LUMO energies were carried out using the Gaussian09 software package^44^. All free linkers were geometrically optimized using a PBE0 functional, before an HSE06 frequency calculation was performed on the optimized structure. A def2svp basis was used, with tight convergence and a fine density grid. A thermodynamic cycle of solvated deprotonation was used to determine the pK_a_s^24^, from the sum of thermal and electronic free energy values of the HSE06 calculations. A value of −110.2 kcal/mol was used for the solvation energy of H_3_O^+^. Remaining solvation energies were determined through by invoking implicit solvent model.

## Supplementary information

Supplementary Information

## Data Availability

All geometries and output files created for this study are available on FIGshare (10.6084/m9.figshare.21741365).
