# Supplementary material for: Ligand field tuning of d-orbital energies in metal-organic framework clusters
Source: Commun Chem. 2023 Apr 12;6:67. doi: 10.1038/s42004-023-00863-z (PMC10097619; doi:10.1038/s42004-023-00863-z)
Supplement: Supplementary file 1 — Supplementary Information [file 42004_2023_863_MOESM1_ESM.pdf]

Supporting Information for

Ligand field tuning of *d*-orbital energies in metal-organic framework clusters

Brian G. Diamond, Lillian I. Payne, and Christopher H. Hendon
Department of Chemistry and Biochemistry, University of Oregon, Eugene, OR, 97403 USA.

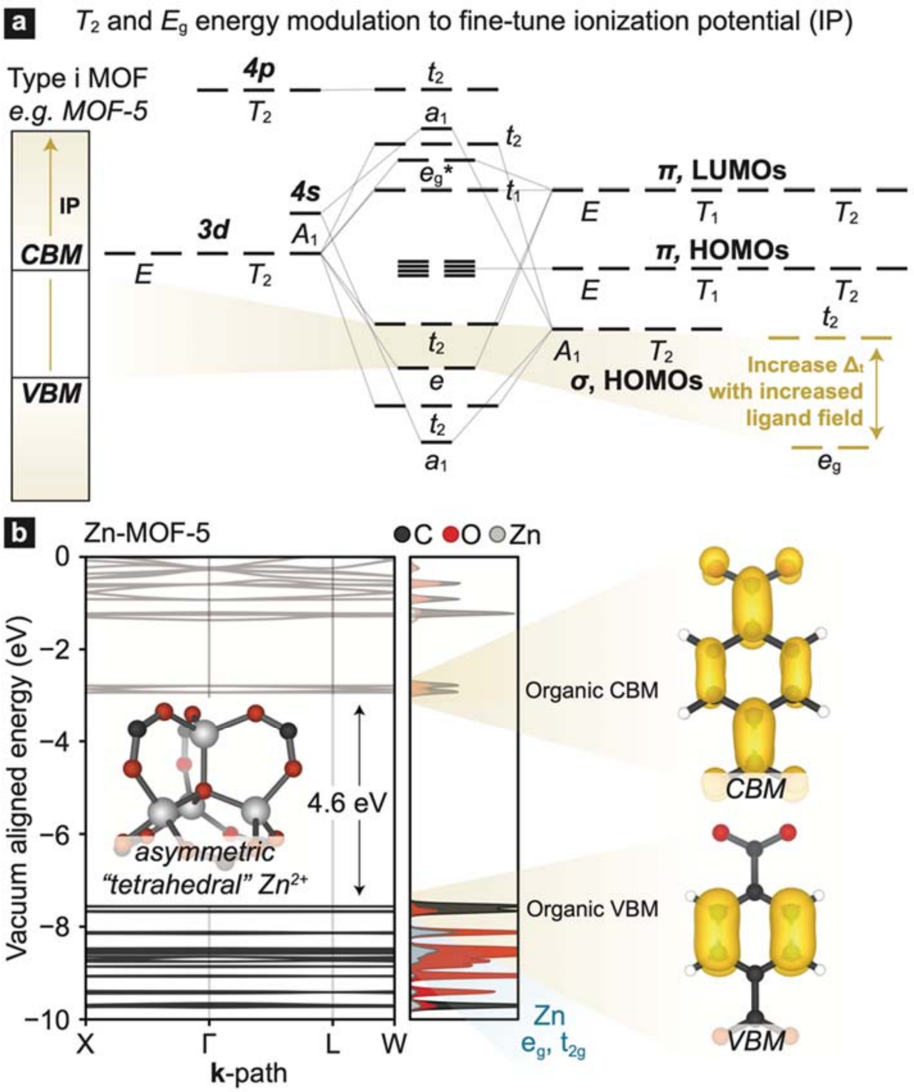

Figure S1. Zn-MOF-5 features pseudo-tetrahedral Zn^{2+} ions. In complex MOF clusters, such as that of MOF-5, **a)** the idealized tetrahedral splitting diagram will depend on the orbital filling of the ligand, but in most MOFs the π-system will dictate the HOMO. **b)** the electronic band structure of MOF-5 reveals that the Zn d-states sit close to the valence band maximum, but below ligand-centered states.

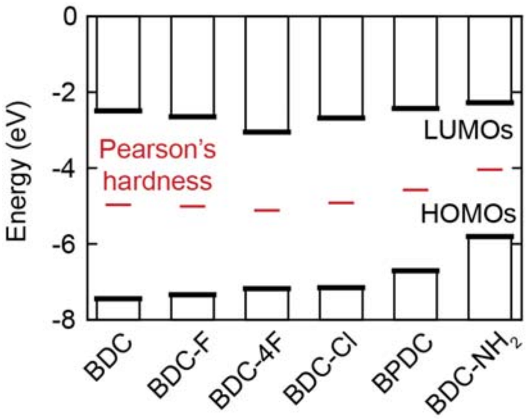

Figure S2. Functionalized benzene dicarboxylate electronic structure and Pearson hardness. Energies are computed using the molecular DFT protocol discussed in the main text.

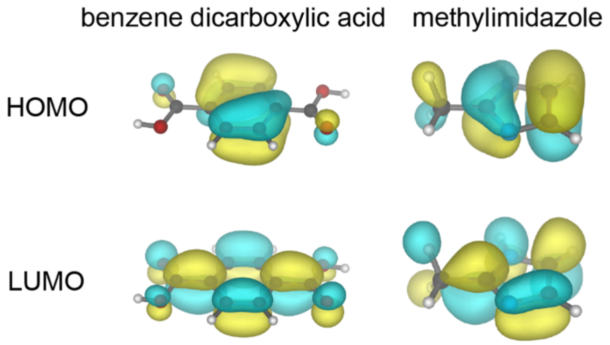

Figure S3. Unfunctionalized linker HOMO/LUMO isosurfaces. Plotted at 0.01 eV/Å^3.

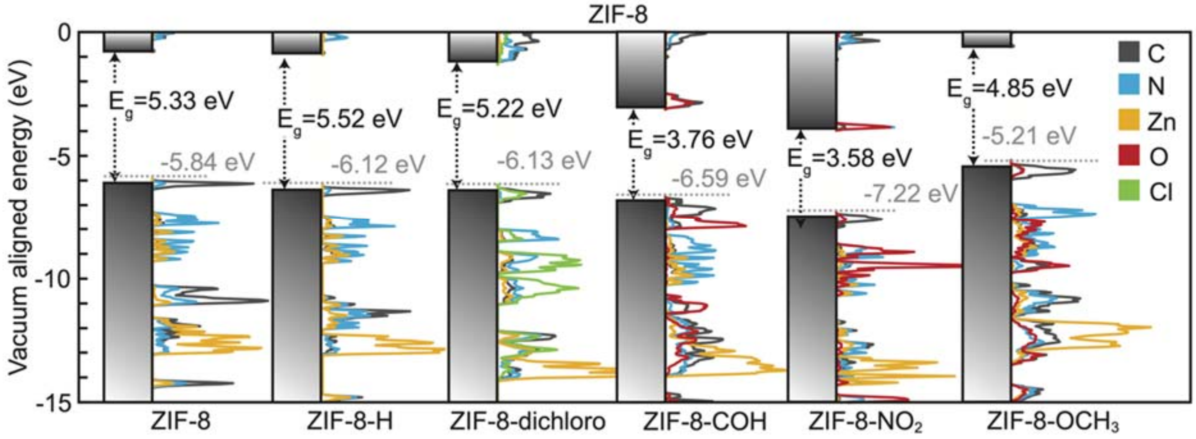

Figure S4. Vacuum-aligned density of states of ZIF-8 and all analogue structures that were studied. The Zn(II) states appear in the conduction band. The orbitals we monitor are highlighted.

Table S1. Electronic band gaps for functionalized MOFs.

Material	Band Gap (eV)
ZIF-8	5.33
ZIF-8-H	5.52
ZIF-8-NO_2	3.58
ZIF-8-2Cl	5.22
ZIF-8-COH	3.76
ZIF-8-OCH_3	4.85
MIL-125	3.78
MIL-125-NH_2	2.34
MIL-125-Cl	3.37
MIL-125-F	3.67
MOF-5	4.64
MOF-5-Cl	4.10
MOF-5-F	4.32
MOF-5-4F	5.38
MOF-10	3.78
MOF-14	3.21
UiO-66	4.15
UiO-66-NH_2	2.79
UiO-66-Cl	3.70
UiO-66-F	3.92
UiO-67	3.64
